# Altered spontaneous brain activity in chronic smokers revealed by fractional ramplitude of low-frequency fluctuation analysis: a preliminary study

**DOI:** 10.1038/s41598-017-00463-7

**Published:** 2017-03-23

**Authors:** Chao Wang, Zhujing Shen, Peiyu Huang, Hualiang Yu, Wei Qian, Xiaojun Guan, Quanquan Gu, Yihong Yang, Minming Zhang

**Affiliations:** 1 Department of Radiology, the Second Affiliated Hospital, Zhejiang University School of Medicine, Hangzhou, China; 2 Department of Psychiatry, the Second Affiliated Hospital, Zhejiang University School of Medicine, Hangzhou, China; 3 Neuroimaging Research Branch, National Institute on Drug Abuse, National Institutes of Health, Baltimore, MD 21224 USA

## Abstract

Although a substantial body of previous functional magnetic resonance imaging (fMRI) studies have revealed different brain responses to external stimuli in chronic cigarette smokers compared with nonsmokers, only a few studies assessed brain spontaneous activity in the resting state in chronic smokers. The aim of this study was to investigate alterations of brain activity during the resting state in chronic smokers using fractional amplitude of low-frequency fluctuation (fALFF). In the present study, 55 smokers and 49 healthy nonsmokers were included. All the subjects underwent resting-state fMRI scans and the data were analyzed by the fALFF approach. The smokers showed significantly decreased fALFF in the left precuneus, right inferior temporal and occipital gyrus(ITG/IOG), while significantly increased fALFF in the right caudate. Subsequent correlation analysis revealed that the fALFF values of the left precuneus and right ITG/IOG were positively correlated with years of smoking across the smokers. This resting-state fMRI study suggests that the changed spontaneous neuronal activity, as reflected by the fALFF, in these regions may be implicated in the underlying the pathophysiology of smoking.

## Introduction

Cigarette smoking, one of the biggest threats to world health, is responsible for 6 million preventable deaths in 2011^1^ and is estimated to cause 8.3 million deaths by the year 2030^2^. Nicotine is primarily responsible for the highly addictive properties of cigarettes. Many functional magnetic resonance imaging (fMRI) studies have been performed to examine the effects of acute nicotine administration in smokers and non-smokers. A common finding from acute administration of nicotine/smoking is the globally reduced brain activity^3^. However, only a few studies^4–7^ reported alterations of regional spontaneous activity during resting state in chronic smokers. Resting-state functional magnetic resonance imaging (rs-fMRI) has recently been suggested as an important tool to explore the pathophysiological mechanisms underlying psychiatric and neurological diseases^8^.

The amplitude of low-frequency fluctuation (ALFF) of the blood-oxygenation level-dependent approach is effective and powerful for examining disease-related local brain activity in the resting state^9^. ALFF has been used in the studies of neuropsychiatric diseases, such as attention-deficit/hyperactivity disorder^9^, major depressive disorder^10^, Alzheimer’s disease^11^, and schizophrenia^12^. Although ALFF appears to be a promising method for detecting regional signal changes of spontaneous brain activity, certain cisternal areas have also shown significantly higher ALFF which is likely due to physiological noise. Thus, the fractional ALFF (fALFF) approach was developed to selectively suppress the noise in signals from nonspecific brain areas and thereby significantly improve the sensitivity and specificity of detecting spontaneous brain activity^13^. Using fALFF on 20 smokers and 19 nonsmokers, Chu *et al*.^6^ observed that fALFF was higher in the left middle occipital gyrus, left limbic lobe and left cerebellum posterior lobe but lower in the right middle frontal gyrus, right superior temporal gyrus, right extra nuclear, left postcentral gyrus and left cerebellum anterior lobe in smokers compared to nonsmokers. Recently, a fALFF study on 27 young adult smokers revealed that smokers had higher fALFF values in the right caudate^7^. The aim of the present study was to further investigate the fALFF of spontaneous brain dynamics in chronic smokers with a relatively large sample size.

## Results

### Demographic information

The results were from 55 smokers and 49 nonsmokers. Smokers and nonsmokers did not significantly differ in age (smokers mean = 39.4, SD = 6.9 years; nonsmokers mean = 37.3, SD = 8.0 years; *t* = 1.437, *p* = 0.154), gender (smokers: male/female = 55/0; nonsmokers: male/female = 49/0; *p* = 1.000) or handedness (smokers: right/left = 55/0; nonsmokers:49/0; *p* = 1.000), though there was a difference in years of education (smokers mean = 13.6, SD = 2.6 years; nonsmokers mean = 16.2, SD = 4.5 years; *t* = −3.566, *p* = 0.001). Therefore, years of education were included as a covariate in later analyses. Detailed demographic information and tobacco use parameters for smokers and non-smokers are summarized in Table 1.

**Table 1:** Demographic characteristics and tobacco use parameters of smokers and non-smokers.

Demographic variables	Smokers	Non-smokers	*P* values
N	55	49	—
Age, years, mean ± SD	39.4 (6.9)	37.3 (8.0)	0.154
Range, years	26–54	25-56	—
Gender (male/female)	55/0	49/0	1.000
Education years, mean ± SD	13.6 ± 2.6^a^	16.2 ± 4.5	0.001
Handedness, right/left (n)	55/0	49/0	1.000
Age at start of smoking, mean ± SD	20.4 ± 5.1	—	—
Smoking initiation age range (years)	12–43	—	—
Smoking years, mean ± SD	19.0 ± 6.1	—	—
Range, years	8–38	—	—
Cigarettes per day	23.5 ± 10.2	—	—
Range, cigarettes per day	10–60	—	—
FTND scores	5.2 ± 2.1	—	—
Range, FTND scores	1–10	—	—

^a^Significantly different from control group, p < 0.01. *FTND* Fagerström test for Nicotine Dependence. Two-sample two-tailed t tests were used for age and years of education comparisons between smokers and non-smokers. Chi-squared test was performed for gender and handedness comparison between smokers and non-smokers.

### fALFF results

**Figure 1:**
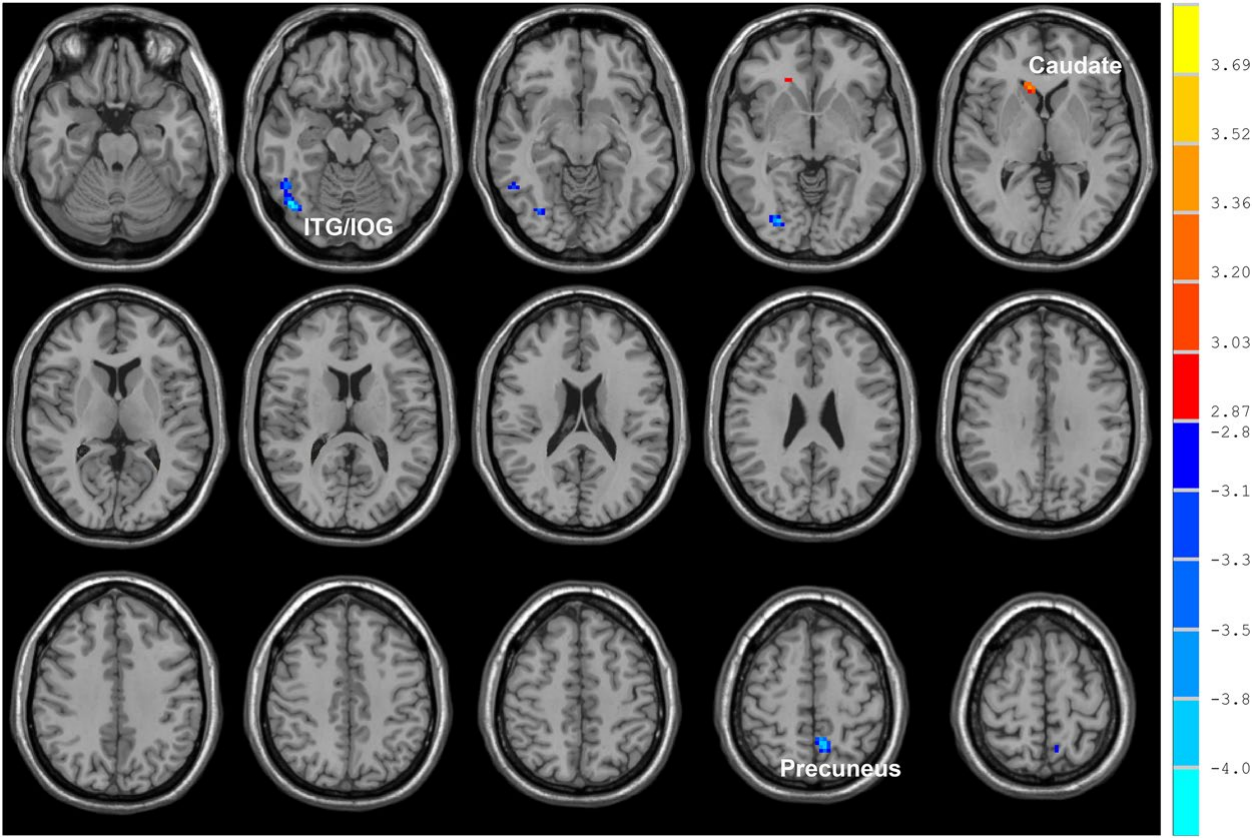
Brain areas of fALFF difference between smokers and nonsmokers. Areas in blue colors are brain regions where fALFF was significantly decreased in smokers compared with nonsmokers. The areas with decreased fALFF were the left precuneus, right ITG and IOG. Areas in red colors show significantly increased fALFF in smokers compared with nonsmokers. The area showing increased fALFF was the right caudate.

**Figure 2:**
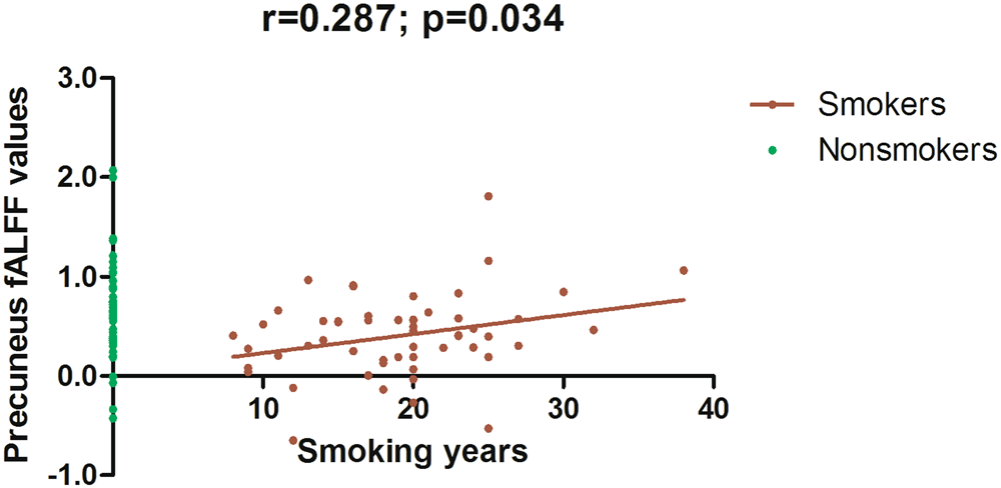
The fALFF values of the left precuneus were positively correlated with smoking years across the smokers (r = 0.287; *p* = 0.034).

**Figure 3:**
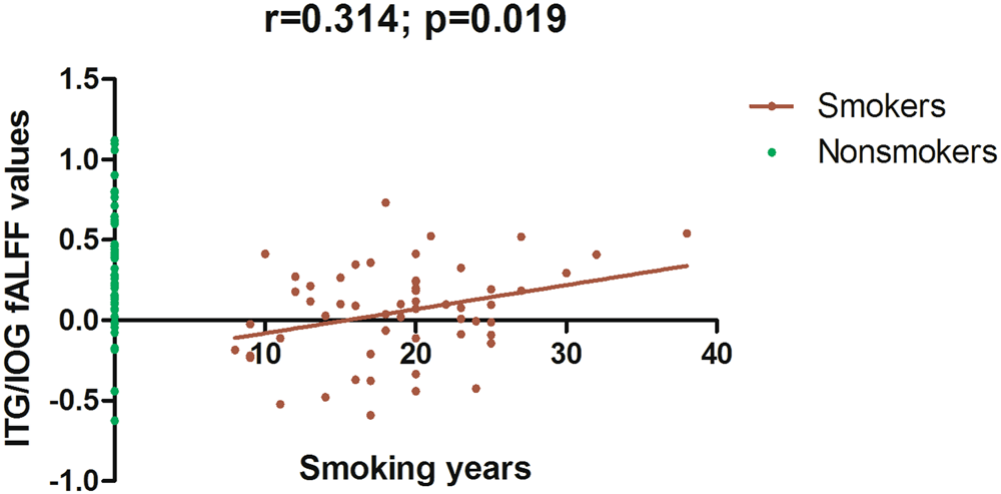
The fALFF values of the right ITG/IOG were positively correlated with smoking years across the smokers (r = 0.314; *p* = 0.019).

Using age, years of education as covariates, in comparison with nonsmokers, chronic smokers showed significantly decreased fALFF in the left precuneus, right inferior temporal (ITG) and occipital gyrus (IOG). In contrast, relative to nonsmokers, chronic smokers showed significantly increased fALFF in the right caudate (as shown in Fig. 1 and Table 2). No other difference in fALFF was observed between the two groups. Subsequent correlation analysis revealed that the fALFF values of the left precuneus (r = 0.287, *p* = 0.034; as shown in Fig. 2) and right ITG/IOG (r = 0.314, *p* = 0.019; as shown in Fig. 3) were positively correlated with smoking years across the smokers (as shown in Table 3). However, there were no correlations between the neuroimaging findings and the other cigarette smoking measures, including the cigarettes per day, FTND and age at start of smoking (as shown in Table 3).

**Table 2:** Brain areas with altered fALFF between smokers and nonsmokers.

Brain areas	Side	MNI coordinates (cluster maxima, mm)			Peak T values (voxels)	Cluster size
		X	Y	Z		
Smokers < non-smokers						
Precuneus	L	−9	−54	57	−3.93	34
ITG/IOG	R	45	−69	−15	−4.20	78
Smokers > non-smokers						
Caudate	R	12	24	3	3.45	27

The result threshold was at P < 0.005, and only clusters exceeding a size of 27 voxels are reported. All coordinates are given in Montreal Neurological Institute (MNI) space. ITG: inferior temporal gyrus; IOG: inferior occipital gyrus; L: left; R: right.

**Table 3:** Correlation analyses between the neuroimaging findings and cigarette smoking measures among 55 smokers.

	Smoking years	Cigarettes per day	FTND	Age at start of smoking
Caudate fALFF values				
Pearson correlation	−0.109	−0.005	0.037	−0.058
Sig. (2-tailed)	0.429	0.971	0.787	0.672
Precuneus fALFF values				
Pearson correlation	0.287*	0.173	0.188	0.000
Sig. (2-tailed)	0.034	0.206	0.169	0.999
ITG/IOG fALFF values				
Pearson correlation	0.314*	−0.008	−0.009	−0.012
Sig. (2-tailed)	0.019	0.953	0.945	0.930

*Significant correlation at the level of 0.05 (2-tailed); *FTND* Fagerström test for Nicotine Dependence; ITG: inferior temporal gyrus; IOG: inferior occipital gyrus.

## Discussion

Many neuroimaging studies have sought to identify the effects of chronic cigarette smoking on structural and functional alterations in the brain. Structural MRI studies on smokers have found that chronic cigarette smoking is associated with gray matter deficits in the frontal, subcortical, parietal, occipital cortices, and the cerebellum^14^. Moreover, diffusion tensor imaging (DTI) studies have demonstrated altered fractional anisotropy in the prefrontal white matter, cingulum, corpus callosum in smokers^14^. Abnormal regional brain activity has been revealed in mesolimbic dopamine reward circuits, including the ventral tegmental area, nucleus accumbens, hippocampus, amygdala, cingulate, thalamus, striatum, and prefrontal cortex, as well as visuospatial attention circuits, including the dorsolateral and ventrolateral prefrontal cortex, parietal cortex, and extra-striate visual cortex in smokers^15, 16^. Furthermore, smokers also demonstrated changes in functional connectivity between the dorsal anterior cingulate cortex and ventral striatum^17^. Recently, several studies^4–7^ reported alterations of brain spontaneous activity during resting state in chronic smokers with a relatively small sample size. However, little is known about the chronic effects of cigarette smoking on the resting state spontaneous activity with a relatively large sample size, especially less was known about the association between the resting state abnormalities and cigarette smoking measures, which was very important to improve the understanding of the neural mechanism in smokers. In this study, compared with 49 healthy controls, 55 chronic smokers showed significantly decreased fALFF in the left precuneus, right ITG/IOG, which suggested that, during resting state, neural function was less active than nonsmokers in these brain regions. In contrast, smokers showed significantly increased fALFF in the right caudate, which suggested that, during resting state, neural function was more active than nonsmokers in this brain region. In addition, the fALFF values of left precuneus and right ITG/IOG were positively correlated with smoking years in smokers. Our findings provided insights into the effects of chronic cigarette use on spontaneous brain activity and the pathophysiological mechanisms of chronic cigarette smoking.

This study observed decreased fALFF for chronic smokers in the left precuneus, which was positively correlated with smoking years in smokers. A recent meta-analysis of fMRI studies of smoking cue reactivity found a reliable cue reactivity effect in the precuneus^16^. The precuneus is a part of the superior parietal lobule, which is involved in planning and executing functions^18^. A meta-analysis demonstrated that parietal lobule was found to be related to the reward-circuit^19^, suggesting that the parietal cortex could be involved in addiction behavior. The precuneus is also an important component of the default mode network (DMN) identified in previous resting-state fMRI studies^20^. The DMN is one of the several brain networks that show spontaneous, synchronous low frequency fluctuations at rest. The DMN is thought to be associated with stimulus-independent thought that is detached from the external environment, and implicated in a series of internal self-referential and reflective activity including personal introspection, autobiographical memories, and thoughts about the future^21^. Many previous studies demonstrated reduced activity in the regions within the DMN after acute administration of nicotine or nicotinic cholinergic agonists in externally cued cognitively demanding tasks^22–25^. In addition, the effects of chronic cigarette smoking on DMN function have been investigated in many studies. A recent resting-state fMRI (rs-fMRI) study by Wu *et al*. revealed significantly decreased spontaneous neural activity in the DMN, such as in the bilateral posterior cingulate cortex/precuneus, bilateral ventral and dorsal medial prefrontal cortex (MPFC), and bilateral angular gyrus^4^. However, another rs-fMRI study by Yu *et al*. showed that heavy chronic smokers exhibited increased spontaneous neural activity in the posterior cingulate cortex relative to healthy controls^5^. One possible causes of this difference that should be noted is the different smoking states of data acquisition. Acute nicotine exposure has been associated with decreased DMN activity in the precuneus, posterior cingulate, and medial orbitofrontal cortex^26^. Wu *et al*.^4^ acquired data on heavy smokers who were allowed to smoke at liberty before scanning, but Yu *et al*.^5^ did not specify the smoking state of smokers. In addition, using brain network analysis, Lin *et al*.^27^ found that heavy smokers had decreased nodal efficiency in brain regions within the DMN, and these participants could smoke at liberty before imaging. In the present study (55 smokers vs 49 nonsmokers), we acquired data on smokers at a state similar to that in Wu *et al*.’s study, and decreased spontaneous neural activity in the left precuneus was found in smokers relative to nonsmokers. Another possible cause of this difference might be the relatively small sample size in Yu *et al*.’s study^5^ (only 16 smokers and 16 non-smokers). By contrast, Wu *et al*.’s study^4^ included a relatively large sample size (31 heavy smokers and 33 nonsmokers). Taken together, these studies suggested that cigarette smoking affected spontaneous brain activity in the DMN, including the precuneus, which is associated with self-referential and emotional processing. Furthermore, smokers showed reduced fALFF in the right ITG/IOG, which was positively correlated with years smoking in smokers. The temporal lobe is the olfactory and auditory center, which are known to take part in resisting smoking craving, such as self-talk, which was one of several strategies employed to resist craving^28^. The occipital lobe was the visual center and was found to be related to attention processing and the visuospatial analysis of environment^29, 30^. Several functional brain imaging studies reported that occipital areas were activated following smoking or nicotine exposure during visual task performance^31, 32^. Activated occipital areas have been regarded as neural correlates of nicotine-induced attention increase^32, 33^.

Notably, subsequent correlation analysis revealed that the fALFF values of the left precuneus and right ITG/IOG were positively correlated with smoking years in smokers. As mentioned above, compared to nonsmokers, smokers showed decreased fALFF in the left precuneus and right ITG/IOG; therefore, we expected to observe more attenuation in the smokers with longer smoking history. However, individuals with shorter smoking history showed weaker fALFF in the left precuneus and right ITG/IOG, whereas those with longer smoking history showed stronger fALFF which was closer to the fALFF value of the nonsmokers. Similar resting-state functional connectivity (rsFC) study showed widespread rsFC attenuation in the reward circuit in smokers and the rsFC was positively correlated with dependence severity^33^. Moreover, microstructural studies found that the generally higher fractional anisotropy (FA) seen in smokers may reflect an increase in FA that follows the initiation of smoking, but prolonged smoking appears to reverse this effect with a progressive decline in FA with increasing pack-years of smoking^34, 35^. In our opinion, these features might represent neuroplastic changes that develop over time to support the development of neurophysiologic dependence.

In addition, smokers showed increased fALFF in the right caudate. A recent fALFF study also revealed higher fALFF values of the right caudate in young adult smokers. The caudate is a part of the dorsal striatum and a key region of the nigrostriatal dopamine (DA) circuits, which are critical for habit formation^36^ and play important roles in craving and reward processing in addiction^37, 38^. Position emission tomography (PET) studies detected that smoking induced dopamine release in the caudate in smokers, which was significantly correlated with craving ratings^39–41^. Experimental evidence also revealed that the caudate mediated nicotine seeking following smoking abstinence and craving provoked by smoking cues^42, 43^. In addition, a recent study reported that the caudate morphology was correlated with craving measures in smokers^44^. Taken together, these neuroimaging findings indicate the important role of the caudate in chronic smoking.

There are some limitations that should be mentioned. Education levels were not well matched in the two groups. However, education level was set as a covariate of no interest in the group analysis. In addition, in this study, we didn’t evaluate sex effects on outcome measures and the findings may be specific to male smokers, since only the male subjects were recruited in this study as most smokers are male in China.

In conclusion, in the present study smokers, compared to matched nonsmokers, showed significantly decreased fALFF in the left precuneus, right ITG and IOG, while increased fALFF in the right caudate. Furthermore, the fALFF values of the left precuneus and right ITG/IOG were positively correlated with smoking years across the smokers. Our findings provided new evidence on neuroimaging measures and smoking behaviors, which may improve our understanding of the neurobiological underpinning of chronic cigarette smoking. Longitudinal studies will be needed to further investigate the casual relationship between the neuroplasticity and addiction.

## Materials and Methods

### Participants

Fifty-five nicotine-dependent male smokers and 49 age-matched healthy male nonsmokers were recruited via advertisements. The inclusion criteria for smokers were as follows: (1) smoking ≥10 cigarettes per day for ≥2 years, (2) meeting DSM-IV criteria for nicotine dependence, (3) having an afternoon breath carbon monoxide (CO) level >10 ppm, (4) no current smokeless tobacco use, (5) being right-handed, (6) being 22–55 year-old. Additionally, demographic and smoking data were obtained from all participants by a questionnaire prior to scanning. Nicotine dependence levels of smokers were assessed with the FTND^45^. The FTND includes 6 items and produces a score from 0 to 10, with higher scores indicating more severe nicotine dependence. The nonsmokers were identified as those smoking no more than 20 cigarettes in their lifetime with expired CO ≤ 3 ppm. Participants with histories of major illnesses, other substance abuse (besides nicotine), psychotropic medication use, neurological and psychiatric diseases, and systemic diseases (i.e., diabetes or hypertension) were excluded. Additional exclusion criteria for all participants included MRI contraindications such as claustrophobia and metal implants. All the procedures were reviewed and approved by the Institutional Review Boards of the Second Affiliated Hospital of Zhejiang University School of Medicine, and all the procedures were carried out in accordance with the approved guidelines. All subjects provided signed informed consents prior to study participation.

### Image acquisition

All the data were acquired using a 3.0 T GE Signa MR scanner equipped with a birdcage coil. Foam padding and earplugs were used to limit head movement and reduce scanner noise for the subject. During the data acquisition, the subjects were instructed to keep their eyes closed, but not to fall asleep, and to relax their minds and move as little as possible. The functional images were collected axially using an echo-planar imaging (EPI) sequence. The imaging parameters were as follows: repetition time = 2000 ms; echo time = 30 ms; slices = 30; thickness = 4 mm; gap = 1 mm; field of view (FOV) = 240 × 240 mm^2^; resolution = 64 × 64; and flip angle = 80°. The scan lasted for 370 s. Three-dimensional axial Fast Spoiled Gradient Recalled (3D-FSGPR) images were collected using the following parameters: TR/TE = 5056 ms/1.116 ms; Flip angle = 15°; FOV = 24 × 21.6 cm; matrix = 256 × 256; slices = 136; thickness = 1.2 mm; and space = 0 mm. After the scan, the subjects were asked whether they were stayed awake or not during the whole procedure. In the current study, smokers were allowed to smoke as usual prior to scanning to avoid withdrawal symptoms during scanning.

### Fractional amplitude of low-frequency fluctuation (fALFF) analysis

The EPI data were preprocessed with the Dam Processing Assistant for Resting State fMRI (DPARSF) (http://www.restfmri.net/forum/DPARSF) that works with SPM8 (http://www.fil.ion.ucl.ac.uk/spm) on the Matlab 7.5 platform. The first ten volumes of the scanning sessions were removed to allow for the magnetization to reach a steady state and participants’ adaptation to the scanning environment. For each subject, the images were slice-timing corrected and realigned. None of the subjects’ head motion exceeded 1 mm of movement or 1 rotation in any direction. After realignment, all of the data were normalized to Montreal Neurological Institute space, resampled with 3 mm × 3 mm × 3 mm resolution and smoothed with a Gaussian kernel of 6 mm full width.

After the above preprocessing, fALFF images were computed in a similar way to the REST software (http://restingfmri.sourceforge.net) as described previously^17, 18^. First, the time series of each voxel was transformed with a Fast Fourier Transform (FFT) to obtain the power spectrum. Then, the square root was calculated at each frequency of the power spectrum, and the averaged square root was acquired as ALFF across 0.01–0.08 Hz at each voxel. Finally, fALFF was calculated using the ratio of power spectrum of low-frequency (0.01 Hz to 0.08 Hz) to that of the entire frequency range.

The statistical analysis of fALFF was performed using Statistical Parametric Mapping 8 (SPM8, http://www.fil.ion.ucl.ac.uk/spm). To investigate the fALFF differences between smokers and nonsmokers, a two-sample *t* test was performed on the individually normalized fALFF maps in a voxel by voxel manner with age and years of education as covariates of no interest. Multiple comparisons were corrected at a threshold of alpha <0.05 determined by the newest version of AFNI 3dClustSim Program. The following parameters were used: single voxel p = 0.005, cluster size = 27 voxels (729 mm^3^), FWHM = 6 mm, cluster connection radius r = 5 mm. Subsequently, we performed a post-hoc correlation analysis in order to assess whether smoking measures were related to the fALFF properties or not. A Pearson’s correlation analysis was performed to assess the relationship between neuroimaging findings (the fALFF values in regions showing differences between smokers and nonsmokers) and cigarette smoking measures (i.e., smoking years, cigarettes per day, FTND, age at start of smoking).
